# National or population level interventions addressing the social determinants of mental health – an umbrella review

**DOI:** 10.1186/s12889-021-12145-1

**Published:** 2021-11-18

**Authors:** Neha Shah, Ian F. Walker, Yannish Naik, Selina Rajan, Kate O’Hagan, Michelle Black, Christopher Cartwright, Taavi Tillmann, Nicola Pearce-Smith, Jude Stansfield

**Affiliations:** 1 City University London, Northampton Square, EC1V 0HB, London, UK; 2 Hull York Medical School, University of York, Heslington, York, YO10 5DD England; 3 Leeds Teaching Hospitals NHS Trust, Great St George Street, Leeds, LS1 3EX England; 4 School of Health and Related Research, The University of Sheffield, Regent Court, 30 Regent, Sheffield, S1 4DA England; 5 Department of Health Services Research and Policy, The London School of Hygiene and Tropical Medicine, London, WC1E 7H UK; 6 Public Health England, Wellington House, 133-155 Waterloo Road, London, SE1 8UG UK; 7 Bradford Institute for Health Research, Bradford Royal Infirmary, Bradford Teaching Hospitals NHS Foundation Trust, Duckworth Lane, Bradford, BD9 6RJ England; 8 Centre for Global Non-Communicable Disease, Institute for Global Health, UCL, 30 Guilford, London, WC1N 1EH UK; 9 School of Health and Community Studies, Leeds Beckett University, Portland Building, PD519, Portland Place, Leeds, LS1 3HE UK

**Keywords:** Mental health, Public health, Public mental health, Health policy, Social determinants, Intervention, Population health

## Abstract

**Background:**

Social circumstances in which people live and work impact the population’s mental health. We aimed to synthesise evidence identifying effective interventions and policies that influence the social determinants of mental health at national or scaled population level. We searched five databases (Cochrane Library, Global Health, MEDLINE, EMBASE and PsycINFO) between Jan 1st 2000 and July 23rd 2019 to identify systematic reviews of population-level interventions or policies addressing a recognised social determinant of mental health and collected mental health outcomes. There were no restrictions on country, sub-population or age. A narrative overview of results is provided. Quality assessment was conducted using Assessment of Multiple Systematic Reviews (AMSTAR 2). This study was registered on PROSPERO (CRD42019140198).

**Results:**

We identified 20 reviews for inclusion. Most reviews were of low or critically low quality. Primary studies were mostly observational and from higher income settings. Higher quality evidence indicates more generous welfare benefits may reduce socioeconomic inequalities in mental health outcomes. Lower quality evidence suggests unemployment insurance, warm housing interventions, neighbourhood renewal, paid parental leave, gender equality policies, community-based parenting programmes, and less restrictive migration policies are associated with improved mental health outcomes. Low quality evidence suggests restriction of access to lethal means and multi-component suicide prevention programmes are associated with reduced suicide risk.

**Conclusion:**

This umbrella review has identified a small and overall low-quality evidence base for population level interventions addressing the social determinants of mental health. There are significant gaps in the evidence base for key policy areas, which limit ability of national policymakers to understand how to effectively improve population mental health.

**Supplementary Information:**

The online version contains supplementary material available at 10.1186/s12889-021-12145-1.

## Introduction

Recent policy priorities in global mental health have focused on closing the treatment gap: improving the proportion of individuals experiencing a mental health problem who are able to access effective psychiatric treatments locally [1, 2]. This is rightly a policy focus globally, but not alone in importance.

The current global mental health action plan [3] also prioritises the promotion and prevention of mental health. The Sustainable Development Goals (SDGs) commit countries to ‘reduce by one third pre-mature mortality from non-communicable diseases through prevention and treatment and promote mental health and wellbeing’ by 2030 [4]. The recent Lancet Commission on Global Mental Health and Sustainable Development [5] emphasised a lack of progress in most nations. The Commission challenged the international community to address this ‘prevention gap’ in mental health.

There is now good evidence that various social determinants are associated with poor mental health outcomes. Socioeconomic factors such as poverty [6, 7], (un) employment and poor working conditions [8–11] and macroeconomic changes such as recessions [12, 13] are associated with mental health. Housing and homelessness [14–16] are also important factors, as are social capital and the availability of social support [17–20]. Other social determinants associated with mental health include crime [21], violence [22–24], education [25–27], social protection mechanisms such as welfare benefits [28, 29] and access to the natural environment (particularly in urban areas) [30, 31].

Many of these determinants were identified in a recent review of reviews [32] of the social determinants of mental health, which incorporated them into a conceptual model using the SDGs, identifying potential targets for intervention. Whilst the importance of intervention in these areas has been recognised, [33, 34] the effectiveness of national or population level policies addressing these social determinants to improve population mental health has not been systematically analysed [35]. Because of this lack of evidence, it is unclear what policies countries can implement to effectively address population mental health.

We undertook this umbrella review to identify the best available evidence for national or population level policies or interventions that improve population mental health and wellbeing by addressing the social determinants of mental health.

## Methods

### Review question

Umbrella reviews provide a transparent and rigorous approach, using systematic review methodology, to locate, appraise and summarise the highest quality evidence from published systematic reviews on a topic [36]. The protocol for this review is registered on PROSPERO (CRD42019140198). Our research question was: What national or population-level interventions or policies that address the social determinants of mental health have evidence of an effect on mental health and wellbeing?

### Search strategy

We systematically searched several databases (Cochrane Library (Wiley), Global Health (Ovid), MEDLINE (Ovid), EMBASE (Ovid) and PsycINFO (Ovid) using search strategies developed by a Senior Information Scientist (see online Additional file 1 for Medline strategy). We were only interested in contemporary findings that would be applicable for the current context so restricted our search from January 1st 2000 to July 23rd 2019. We also undertook citation searches by checking the references of included studies to identify any further reviews. Grey literature sources were not searched.

Reviews, scoping reviews, systematic reviews and meta-analyses that were published in peer-reviewed journals and met the Database of Abstracts and Reviews of Effects (DARE) [37] criteria for systematic reviews were included. Reviews including before-and-after studies were included. Only reviews in English were included as we had no other technical language skills in the review team.

Reviews were included if they reported national/population level policies or initiatives that had been evaluated at that scale (larger regions/provinces were included if the population was over a million). We placed no restrictions on age, country or sub-population. All sectors that incorporated a social determinant of mental health as recognised by World Health Organisation (WHO) [38] were included (see appendix for search strategy). Reviews needed to report evidence of outcome in respect to positive mental health, wellbeing, reduction in symptoms of mental ill-health, reduction in completed or attempted suicides or prevention of mental health problems. Outcomes were recorded using self-reported wellbeing, screening instruments and diagnostic interviews. Reviews were excluded if they did not include interventions delivered at national/population scale or did not extract mental health outcomes.

### Data collection

EndNote reference management software [39] was used to store and perform an initial screen of the references. After duplicates were removed, pairs of researchers independently screened titles and abstracts against the inclusion criteria. Disagreements were resolved through discussion between pairs with unresolved disagreements arbitrated by a third researcher.

A data extraction form (see additional file 3) was adapted from Naik et al. [12]. Data were double extracted by pairs of reviewers and then checked and arbitrated as in the first stage of screening. Inclusion criteria were based upon the scope and approach of the review. Some reviews included primary studies that were local level and some that were national level. We included these reviews but extracted only the relevant findings from the national/population level studies.

To undertake a quality assessment of each included review we used the Assessment of Multiple Systematic Reviews (AMSTAR 2 - see additional file 2) [40]. Although designed for reviews of healthcare interventions the AMSTAR 2 tool has been used in similar public health umbrella reviews [12, 32]. Only summary findings for mental health outcomes were extracted. All shortlisted reviews were included even if they were assessed as low or critically low quality, with acknowledgement made within the narrative of the need for caution when interpreting these results.

### Data synthesis

Due to the heterogeneity of included studies in terms of methods, outcome measures, topic areas and contexts a meta-analysis was not planned or undertaken. A narrative overview is provided, grouping reviews into topic areas.

## Results

**Fig. 1:**
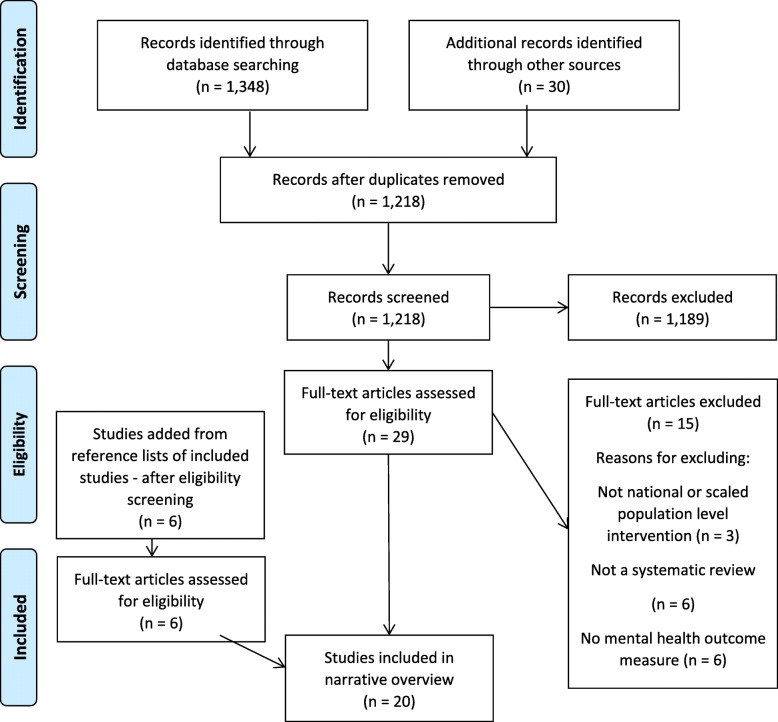
PRISMA Flow Diagram for review screening and inclusion

After removing duplicates, 1218 studies were identified for initial screening. This identified 29 reviews for full text screening, of which 14 systematic reviews met the inclusion criteria. A further 6 reviews were identified from citation searches giving a total of 20 reviews included in this umbrella review (Fig. 1) (see additional files 4 and 5).

The reviews comprised a range of methodologies including meta-analyses and narrative reviews. The majority of reviews focused on high- or upper middle-income countries, and much of the underlying evidence base was made up of observational studies including cohort studies and cross-sectional studies rather than experimental intervention designs. There was some limited use of modelling studies.

Overall, the AMSTAR 2 assessment of quality indicated most reviews were of low or critically low quality, with only four reviews of a high or moderate quality. Common weaknesses included a lack of consideration of quality or bias, an unstructured discussion of these issues, and a range of different structured tools to appraise retrieved studies. Reviews were grouped according to five key domains from a conceptual framework of social determinants of mental health which aligns with the UN SDGs, and thus is relevant to an intent to align intervention evidence with national policy [32] (Table 1).

**Table 1:** Overview of included study category and AMSTAR grading

Conceptual domain as per Lund et al [32]	Relevant social determinant search terms used	Amstar 2 Score for reviews (topic of focus)			
		High	Moderate	Low	Critically low
Demographic	Stigma, prejudice				3 (gender equity)
Economic	Employment, income inequality economic development	3 (welfare)			4 (unemployment insurance or benefits)
Neighbourhood	Housing, violence, crime, community, neighbourhood, volunteer		1 (housing intervention)		1 (housing provision)
Environmental	Conflict, environments, pollution, green/blue spaces, flooding			1 (migration policy)	
Social/Cultural	Perinatal, parenting, school/education, internet, nutrition, sleep, physical activity, lifestyle, drugs, alcohol, social isolation, arts culture,				6 (education and awareness, screening, and restricting means to prevent suicide) 1 (parenting)

### Findings of included studies

### Demographic determinants

We found four reviews which addressed gender policy interventions and their impact on mental health (one of which focused on welfare but looked at gender equity), all of which were of critically low quality.

Length of maternity leave and whether the leave was paid or not was examined by three reviews [41] [42] [43], and included studies of either cross-sectional or longitudinal design. The majority of included studies were from the USA where there was no statutory paid leave; others were from Norway, Lebanon, Canada, Australia where there is paid leave. All three reviews concluded that taking longer and paid leave was associated with better maternal mental health outcomes. Two reviews [42, 43] reported positive benefits from 8 to 12 weeks of paid leave, with another review reporting benefits beyond 12 weeks of maternity leave [43] . All reported greater benefits to mental health or wellbeing with longer leave. The majority of studies assessed results at the individual level, and Aitken et al. [41] reported that no clear conclusion can be made from studies at the population level. There was some evidence to indicate that women experienced improved mental health outcomes when they received extra social support but worse mental health outcomes when their partner did not take any leave.

Borrell et al. [42] found evidence to support the association of policies related to violence against women, economic security, and family planning with better mental health in studies using data from Europe and USA [42]. US states where women had greater reproductive rights reported lower mental health problems at population level [44]. Another review concluded that gender inclusive welfare states (family focused benefits and labour market support for women) improve mental health outcomes in women but don’t necessarily reduce socio-economic inequalities [45]. Dual-earner models, typical of Nordic countries, where both partners contribute to wage earning and caring responsibilities report better mental health outcomes for women. Conversely, market-oriented models which allow market forces to dominate how gender relations are shaped, leaving individuals to find private solutions based on their market resources and family support (typical in the UK) were associated with worse mental health outcomes for women [32, 45]. Dual-earner models appear to support women’s employment while facilitating work life balance with more equitable sharing of domestic work, leading to better health outcomes.

### Economic determinants

Seven reviews of mixed quality (three high and four critically low) looked at economic interventions.

Two high quality reviews looked at conditional benefits such as tax credits and parental benefits tied to employment as examples of welfare to work policies [46, 47]; and concluded there was no effect of these policies on mental health outcomes. A meta-analysis looking at any type of ‘welfare to work’ interventions for lone parents [46] found an initial decline in parental mental health and no change in child mental health with subsequent improvements over time, up to six years following intervention implementation. However, the size of the effect was minimal suggesting this is of questionable importance. They also reported small initial increases in employment and income following the intervention, which disappeared over longer term. As such, mental health impacts did not appear to align with changes in employment and income.

Lucas et al. [48] in a high quality review examined the impact of welfare policies, such as direct cash payments and positive taxation schemes, aimed at socioeconomically disadvantaged families. They reviewed randomised controlled trial (RCT) evidence only and concluded there was no consistent evidence of effect on child mental health or emotional state measured at the individual level [48]. However, policies that are implemented may differ to those that are proposed and the authors concluded that the low monetary value and restrictive conditions placed on receiving such benefits within the included studies could have limited their effectiveness, proposing that this may have been mediated by increased parental stress.

Two critically low quality reviews [49, 50] looked at effects of unemployment insurance (UI) internationally and concluded that more generous UI is associated with better mental health amongst both the unemployed and the employed. O’Campo et al. [50] in a realist review, report that the unemployed do not achieve the same levels of wellbeing as the employed. There was also evidence to indicate that generous UI was associated with re-employment being delayed until towards the end of the benefit period and thus may have negative consequences of disincentivising job-seeking or delaying employment which ultimately would be more beneficial to mental health. The impact of UI on self-rated health was lower in Germany than the US which authors speculated to be due to the receipt of support through other welfare programmes. Regional variations were also found in Spanish youth receiving UI, with both negative and positive mental health impacts. Authors proposed that stigma may account for negative impacts [51]..

McAllister et al. [45], in a review of critically low quality but using studies primarily appraised as high quality and with large samples, found consistent evidence that more generous welfare benefits were associated with reduced socioeconomic inequalities in mental health outcomes. The review also found evidence to the contrary, identifying associations between austerity measures and worse mental health across lower socioeconomic groups and increased suicide rates.

One review of critically low quality assessed the impact of privatisation of public sector utilities [52]. Only three studies were found, which looked at data from the UK and Portugal, and are now 20–30 years old. All three studies suggested detrimental impacts of privatisation on mental health of workers. Authors concluded this was due to job insecurity.

### Neighbourhood determinants

One critically low quality review looked at the provision of mixed income housing (publicly subsidised rental housing development, with deliberate mixing of income groups) and tenant-based rental assistance programmes (subsidised housing for poorer families to live in more affluent neighbourhoods) [53]. Analysis of tenant-based rental assistance programmes suggests an overall 8% median reduction of symptoms of depression and anxiety by the head of household (range 6·5% - 9·5% reduction) based on two studies. The authors concluded that there were too few studies to draw a firm conclusion.

Another review of moderate quality focused on housing interventions [54]. It found evidence of improvements in mental health associated with warmth interventions; some limited and low-quality evidence of improvements in mental health associated with refitting and rehousing interventions; and inconsistent mental health impacts of neighbourhood renewal. McAllister et al. [45] also found evidence that neighbourhood renewal in deprived areas is associated with improved mental wellbeing in women and that area-based initiatives more generally can prevent or reduce social inequalities in mental health.

### Environmental determinants

One review of low quality looked at entry and integration policies for migrants to high income countries [55]. Policies were categorised by generosity: whether migrants’ access to health-promoting resources and opportunities was increased (generous) or limited (restrictive) by the policy. Meta-analysis of policies at ‘entry’ stage suggested that more restrictive entry policies, for example reduced mobility in detainment and use of temporary rather than permanent visas, were associated with an increase in scores for measures of psychological distress, depression, anxiety, or post-traumatic stress disorder [Standardised Mean Difference 0·44 (95% CI 0·13 to 0·75)]. Meta-analysis suggested that more restrictive policies in the integration phase, such as burden of proof for eligibility, cost and long waiting times to access welfare support were associated with increased risk of poor mental health for migrants [OR 1·58 (95%CI 1·03 to 2·42)]. In both cases, risk of bias among the included studies and substantial heterogeneity among the reported results led the authors to conclude with only a low level of certainty. The impacts of integration policies on migrant mental health varied according to the type of policy. The greatest mental health impacts were identified in contexts where migrants are seen as socio-politically separate from their host society and access to welfare support is dependent on labour market participation (exclusionist) [56]. A protective documentation policy, allowing easier and longer-term legal rights to remain in the country was shown to be beneficial for mental health of undocumented migrants in robust and weak studies alike.

### Social/cultural determinants

Five reviews of critically low quality looked at national level suicide prevention programmes and included interventions to improve education and awareness, implement screening for those at risk and restrict access to means [57–61].

#### Restriction to means

Gunnell et al. [57] concluded that following national bans of highly hazardous pesticides in countries such as Sri Lanka, South Korea and Bangladesh, where this was a commonly used method, pesticide suicides fell by between 24 and 40% and overall suicides by 8 to 24% compared to expected rates. Use of restrictive measures rather than bans did not always result in reduced deaths by suicide.

Zalsman et al. [61] found good evidence to support packaging of analgesics, erection of barriers at jump sites; detoxification of gas in catalytic converters in cars; restricting prescription and sales of barbiturates; reducing concentration of caffeine tablets; restricting pesticide availability where this is a prevalent risk factor for reducing suicides.

There were lower rates of suicide when restricting detoxifying domestic gas (19–33% in UK and Switzerland), when restricting access to barbiturates (23% in Australia); and mixed effects related to restricting gun access across a range of countries, with greater impact in high risk populations. Mann et al. [60] reviewed ecological studies suggesting that restricting access to guns was associated with 1·5–9·5% lower suicide rates in the US.

#### Awareness and educational campaigns 

Torok et al. [58] and Zalsman et al. [61] found little evidence to support the use of mass media campaigns as a standalone approach to prevent suicide; however Torok et al. found positive results from studies that looked at mass media campaigns as part of a multi-component approach to suicide prevention. Three multicomponent studies reported reductions in attempted suicide of between 17 and 61%, with one other study reporting reductions in actual suicide deaths.

Zalsman et al. [61] report that RCTs show reduced suicide attempts and ideation following school-based mental health and suicide awareness programmes, with or without combined mental health screening.

Mann et al. [60], reviewed the effects of components of suicide prevention strategies, and found that education of primary care physicians was associated with 22–73% lower annual suicide rates (using cohort data from Sweden, Hungary and Japan). Gatekeeper education is associated with 33–40% lower suicide rates in armed forces populations.

Zechmeister et al. [59] simulated cost effectiveness of scaling up two types of interventions (general awareness and preschool education). They estimated a fall in suicide rates of 57% following general education and 60% following peer support programmes, concluding that both were cost effective: one suicide could be prevented for every 2 dollars on general education and every 4 dollars spent on peer education. Lynch [62] used the ‘Perry Pre-School study’, together with three other similar studies, to develop an economic model and concluded that providing these programmes to just 20% of all 3 and 4 year olds living in poverty in the US could lead to return benefits of the value of between 4 and 9 dollars for every dollar spent on the intervention.

#### Screening

Zalsman et al. [61] concluded that there is insufficient evidence of the benefits of suicide screening in primary care populations for reducing risk of suicide, even when targeted in high-risk populations.

Kato et al.,  [63] in a review of critically low quality, looked at population level family interventions to improve mental health; with a focus on community based parenting programmes. Low quality studies were included and there was no formal quality appraisal. Childrens’ and families’ mental health improved, prevalence of child maltreatment reportedly decreased with positive results on behaviour and mental health problems of children and parental confidence.

## Discussion

**Fig. 2:**
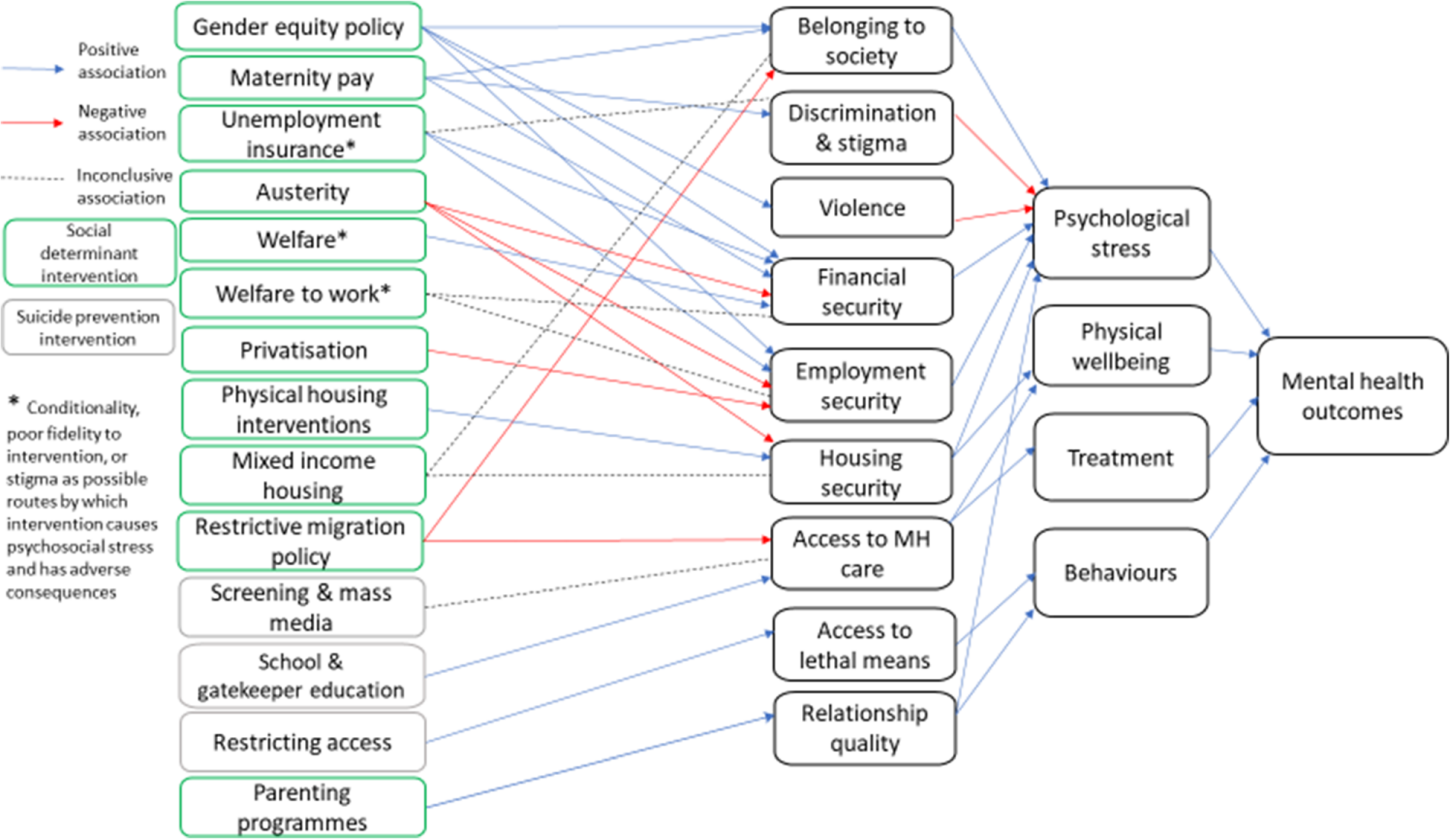
Overview of mechanistic pathways for interventions as proposed by authors of included reviews

In this literature review, we found a broad, but generally low quality evidence base for interventions that address social determinants of mental health at the national or population level. Review level evidence was found across demographic, economic, neighbourhood, environmental, and social/cultural domains of social determinants of mental health as set out by Lund et al. [32], however the majority of the literature was focused in areas of economic intervention and suicide prevention. High level findings are outlined in Table 2. In Fig. 2, an overview of mechanistic pathways for the impact of national/population level interventions addressing social determinants of mental health has been synthesized from the intervention mechanisms proposed (although not necessarily tested) by authors of the included systematic reviews. The majority of pathways involve altering psychological stress experienced through exposure to social determinant risk factors for poor mental health, in particular financial/employment security and belonging to society.

**Table 2:** Overview of intervention effects and pathways

Domain	Intervention	Mechanistic pathway (as stated in review)	Overall evidence on MH outcomes (positive effect, negative effect, no effect, inconclusive, mixed effect, suggestive but inconclusive, insufficient evidence)	Strength of Evidence: AMSTAR 2 grading (number of reviews)
**Demographic**	Paid maternity leave	Reduced stress associated with transition and adapting to multiple roles, new identities, financial strain.	positive	critically low (3)
	Gender equity policies e.g. reproductive rights/ family planning, policies related to violence against women, family supportive employment	Reduction in stress, discrimination, violence, financial difficulties, poverty, double burden of work and caring/household tasks, or time pressure	positive	critically low (2)
**Economic**	Generous welfare benefits	Welfare state interventions alleviate financial pressures on women particularly, reducing gender inequalities in mental health outcomes	positive	critically low (1)
	Austerity	Reduced social spending limits potential to alleviate psychosocial stress related to health and social care, employment, housing and family needs for those seeking to access state social support	negative	critically low (1)
	Benefits for families in poverty	Parental psychosocial stress as the link between low income and child mental health outcomes	no evidence of effect	high (1)
	Welfare to work	Increase in relative income position reduces psychological stress. Also combined consumption and status effects, where income effects on health are mediated by material conditions and in turn social exclusion, and thereby through both physical and psychological mechanisms. Parental stress in turn impacts on child mental health.	no evidence of effect	high (2)
	Unemployment insurance	Generous UI increases financial security which increases psychological wellbeing. Effects on employed are through reducing job insecurity as a chronic psychosocial stressor. Potential negative mechanisms through disincentive to work, stigma.	positive	critically low (2)
	Privatisation	Increases stress through increased job insecurity	negative	critically low (1)
**Neighbourhood**	Mixed income housing in low income neighbourhoods	Positive impacts on MH through improvement in neighbourhood physical and social conditions; negative impacts through disruptions of social ties and social deterioration in receiving neighbourhoods.	insufficient evidence	critically low (1)
	Tenant based rental assistance	As above	inconclusive but suggestive positive	critically low (1)
	Warmth related housing improvements	Inhibiting a key intermediary between poverty and poor health. Qualitative data revealed links via increased thermal comfort, increased space, reduced noise and increased housing satisfaction.	positive	moderate (1)
	Physical housing improvements – rehousing/retrofitting, rehousing from slums, housing led neighbourhood renewal	As above	mixed	moderate (1)
**Environment**	Entry and integration policies for migrants to high income countries	Exclusionist contexts were worse for mental health than assimilationist (where migrants are afforded citizenship but encouraged to conform with host society norms) and the best levels of mental health were associated with integrationist contexts (where migrants are accepted in and afforded rights within the new community.)	negative	low (1)
**Social/Cultural**	Family interventions (inc parenting programmes)	Behavioural problems are likely to lead to secondary mental health problems, such as depression	positive	critically low (1)
	Restriction of access to lethal means	Restriction of access prevents successful completion of suicide but not mental distress	positive	critically low (3)
	School based MH education	Education and awareness among patients and/or physicians, leading to increasing appropriate anti-depressant prescribing, lower rates of untreated major depression and lower suicide	positive	critically low (1)
	Gatekeeper education	As above	positive	critically low (1)
	Screening	Increased identification and treatment of MH problems	insufficient evidence	critically low (1)
	Mass media campaigns	To change behaviour by affecting decision-making processes at the individual level through message promotion, potentially before crisis occurs.	no effect	critically low (1)

### Key findings

There are very few high quality reviews on the impacts of tackling the social determinants on mental health outcomes [35]. The majority of the reviews we included were rated to be of critically low quality. Most reviews included only a small number of studies with mental health outcomes. The majority of evidence from the primary studies was from higher income settings and may not be translatable elsewhere. Whilst some reviews were able to include RCTs, many were reliant on quasi-experimental designs with or without controls (including natural experiments), or used data from cohort and cross sectional studies including interrupted time series analyses and ecological studies.

There are several specific findings from this umbrella review.

First, we found high quality evidence to suggest that more generous welfare benefits may reduce socioeconomic inequalities in mental health outcomes, with evidence to the contrary, showing that austerity measures are likely to have detrimental impacts on mental health.

Second, a wide range of lower quality evidence suggests that social policies were associated with improved mental health outcomes. These included more generous unemployment insurance (which benefits both the employed and unemployed), warmth focused housing interventions, neighbourhood renewal, paid parental leave, gender equality policies, community based parenting programmes, and less restrictive migration policies. Restriction of access to lethal means and multilevel awareness and education programmes were associated with reduced suicide risk.

Third, there was high quality evidence that found no evidence of effect of conditional welfare-to work benefits on adult mental health outcomes. This may be because of the stress induced by the conditions attached to qualify for support or the benefits themselves being too small or restricted to improve material or psychological conditions of poverty. There was also no evidence of effect on child mental health outcomes.

Despite using several search terms, we found very little evidence on social, cultural and community based interventions, delivered at population level and their impact on mental health apart from those linked to suicide prevention. It is possible that these interventions, whilst widely used are under-evaluated or not evaluated specifically for mental health outcomes at a population level.

### Our findings in the context of the literature

We address a critical gap in the literature, by examining the systematic review evidence base around the impacts of gender equality policies on women’s mental health. This review found that evidence in this area was concentrated in high income countries, with little evidence found outside of US and Europe.

The findings of this review are in keeping with the WHO recommendations on national level action on social determinants of health [64, 65] which include targeting families of people with mental disorders in poverty alleviation programmes and social welfare for the unemployed. Lund et al. [28] discuss the association between poverty and mental disorders and that employment is protective of mental health. It is therefore unsurprising to find support for national welfare policy as an effective intervention for mental health; alongside indirect effects of unemployment insurance on the employed, likely through increased sense of security. This review also highlights need for more research and consideration of the conditionality, stigma and context of benefits and how these impact on effectiveness of interventions in these areas.

In the current context of the COVID-19 pandemic as well as numerous current natural disasters, violence and conflict, many linked to climate change, the need for national level intervention to address environmental determinants of mental health is clear, and the gap in literature on this specific domain is concerning.

We found little evidence for interventions in the neighbourhood domain, with warmth related housing interventions showing some promise. This is at odds with international policy recommendations for action to promote access to employment, health care, housing, and education to improve mental health outcomes [32, 64, 65]. This disparity may be due to the focus of our search being national/population level interventions, and thus not capturing relevant literature on neighbourhood and community-level interventions that may be delivered at scale. Addressing the factors associated with successful implementation and scale-up of evidence-based interventions, applying principles of implementation science [66], is important to ensure benefits of neighbourhood level interventions are maintained when delivered at scale.

Whilst the quality of Kato et al’s [63] review was critically low; the existing extensive evidence base on local parenting interventions and the impact of parenting on mental health [67] supports the case for scaled up community parenting intervention.

The WHO recommends nations should have suicide prevention policies in place [3]. This review supports the use of tailored approaches to means restriction and multilevel awareness and education campaigns; but does not find convincing evidence on use of screening or standalone mass media campaigns.

The WHO European health equity framework [68] also highlights income security and social protection; decent living conditions; social and human capital and decent work and employment conditions as key areas of action. To inform national mental health policies, policy makers require research that evaluates the impacts of strategies that address the social determinants and factors that affect their implementation [69]. In regard to mental health and wellbeing inequity, this review finds limited evidence of how to achieve this; with some evidence supporting effective social protection and gender/family policy. The lack of evidence may not only be due to the absence of policy, but also the inadequate funding to implement or evaluate the relevant policies that do exist. This review confirms this research gap and the need for further natural experiments and evaluations of non-health policies that address the social determinants of mental health.

Finally, there is a case to align approaches to tackling social determinants of mental and physical health, given recent literature detailing psychosocial pathways by which social determinants impact on both physical and mental health [70]. Bambra et al. [35], looking at social determinants of health overall, without specifying population level delivery, found evidence of impact across both physical and mental health across rental assistance programmes so that low income families can choose where to live; improving environmental housing interventions; employment changes that affected shift patterns, and job control or security. We did not map against combined mechanistic pathways for physical and mental health, but this could be a useful focus for research going forward.

### Limitations of review

Our focus on national/population level interventions, may have excluded relevant interventions occurring at lower than national level. Future reviews should focus on local or community level interventions which address social determinants of mental health with potential to scale at population level.

While we used a recognised tool to assess quality of reviews, the AMSTAR 2 may not be most appropriate for complex public health interventions. Review tools such as the AMSTAR 2, developed in relation to healthcare research, are conceptually framed and weighted towards a hierarchy of evidence that prefers RCT and meta-analytic synthesis and assumes that they are possible. A rigorous and well considered public heath systematic review that contains different forms of evidence may struggle to score highly on this criterion.

Refining the search to systematic review level evidence only, may also have missed individual studies with good evidence that have not yet been synthesized at review level. We relied on variable methods used in the included systematic reviews for appraisal of quality and synthesis of primary studies.

There are methodological challenges associated with evaluating social interventions delivered at population scale and which can be distal to the mental health outcome, forming complex pathways to impact.

Methods employed in primary research studies limit the strength of findings. Quasi-experimental and observational studies have limited power to explain causality but were commonly used; likely due to the scale of intervention, population of interest, ethical considerations, policy governance (the extent to which researchers are able to influence it or speed and scale of implementation) or availability of data. Bias was introduced in many studies by sub-optimal measurement (such as reliance upon retrospective self report) of mental health and intervention related factors, and not including all relevant populations in the dataset.

To enhance the strength of future review level findings, primary evaluations of mental health policies and interventions should be planned in advance, before implementation of new policies/interventions, to include robust measurement of mental health status longitudinally with samples that are representative of diverse populations. Where experimental designs are not employed, observational studies can employ analytical methods designed to overcome methodological limitations. One approach is to create control groups within one population dataset, for instance employing a difference-in-differences analysis to compare impacts of policies on different subgroups of the population [71]. Another approach is to measure potential confounders and adjust for them in analysis through methods such as stratification, matching, or standardization.

Fidelity is difficult to achieve as most of these interventions are implemented in varied and broad contexts, and are influenced during implementation by multiple contextual factors, many of which are not easily measured. A key example of implementation related factors is the conditionality of welfare interventions (an aspect of implementation rather than core component of the intervention) as potentially mitigating mental health benefits of increased income [48]. Renahy et al. [49] also found variability in mental health impacts of unemployment insurance, suggesting societal stigma as a relevant contextual factor. The majority of included reviews discussed possible impacts of context and implementation but did not directly evaluate this at review level.

Authors of included reviews highlighted that future studies should take implementation and contextual factors into account during design and analysis to enable more comprehensive evaluation. This could include a priori identification of likely relevant implementation or contextual factors which can be collected and subsequent sub-group, sensitivity or effect mediation/moderation/interaction analyses carried out as appropriate. Other methodologies can enable investigation of context-related clustering without measuring factors directly. Multilevel analyses of cross sectional or longitudinal data allow measurement of change across individuals and the wider social contexts in which they live, including analysis of cross level interactions and associations, and variation of effect for target population and the wider population [72]. In ecological studies, adjustment for time and country specific effects can be made [73]. At review level, realist approaches should be considered to better draw out population and context related factors which affect effectiveness of policies or interventions [74].

Clarity on mechanistic pathways can help understand how these interventions work most effectively and what is needed to support this in implementation, but evaluation designs often do not incorporate this. None of the included reviews collected outcomes at points along the proposed mechanistic pathway, and it is unclear if this was done in primary evaluations. The mechanistic pathways set out in Fig. 2 can act as a starting point for developing causal frameworks for more comprehensive testing and evaluation of future interventions. Complex system approaches and associated frameworks provide routes to better integrate evidence related to intervention, mechanism and context [75].

## Conclusion

This umbrella review has identified a small and overall low quality evidence base for national level interventions addressing the social determinants of mental health.

The most robust evidence base centred on welfare and employment support, which national policymakers should consider for implementation. Other national level interventions that are indicated in this review for policy consideration are gender equity policy, less restrictive migration policy, restricting means/ multi-component campaigns for preventing suicide and at scale delivery of community parenting interventions.

There is a need for better quality primary research and improved reporting in systematic reviews that focus on impacts of conditional benefits, neighbourhood renewal or mixed income housing on mental health. No systematic review level evidence was found relating to national strategies for violence reduction, addressing environmental hazards, community services, age and ethnicity inclusive policies and their impact on mental health. There is a wider evidence base of locally evaluated interventions that may be scaled but it is important for these to be evaluated to assess effectiveness when implemented at national/population level.

## Supplementary Information

**Additional file 1.** Search strategy used in OVID Medline

**Additional file 2.** AMSTAR 2 checklist tool.

**Additional file 3.** Data extraction tool

**Additional file 4. **Excluded studies at full text screening stage (with reason)

**Additional file 5.** Summary table of included studies.

## Data Availability

The datasets generated and/or analysed during the current study are available from the corresponding author on reasonable request.
