# Supplementary material for: National or population level interventions addressing the social determinants of mental health – an umbrella review
Source: BMC Public Health. 2021 Nov 18;21:2118. doi: 10.1186/s12889-021-12145-1 (PMC8599417; doi:10.1186/s12889-021-12145-1)
Supplement: Supplementary file 1 — Additional file 1. Search strategy used in OVID Medline [file 12889_2021_12145_MOESM1_ESM.docx]

**Umbrella Review Social Determinants of Mental Health**

**Ovid Medline Search Strategy**

1. (social determinant* or social inequalit*).tw,kw.

2. social gradient*.tw,kw.

3. social status.tw,kw.

4. disadvantaged.ti.

5. (race or racial or ethnicity).ti.

6. occupational status.tw,kw.

7. social support.ti.

8. socioeconomic*.tw,kw.

9. (unemploy*or employment or job or worker* or workplace or employer or welfare or income or economic development or pay or salary or wage).tw,kw.

10. deportation.tw,kw.

11. social class*.tw,kw.

12. living condition*.tw,kw.

13. (hous* or accommodation or dwelling).tw,kw.

14. heating.ti.

15. overcrowding.tw,kw.

16. (diet* or nutrition* or food).ti.

17. (discrimination or stigma* or anti-stigma or prejudice).tw,kw.

18. (family or familial).ti.

19. (parent* or perinatal or prenatal or natal).tw,kw.

20. (breastfeeding or breast feeding).ti.

21. (abuse or maltreatment).tw,kw.

22. neglect*.ti.

23. ((neighbourhood adj safe*) or (neighborhood adj safe*)).tw,kw.

24. population density.tw,kw.

25. (violen* or anti-violence or crime or crimes or criminal or conflict or peace or peace-keeping).tw,kw.

26. homeless*.tw,kw.

27. (bullying or anti-bullying).tw,kw.

28. (school or educ* or college or training).ti,kw.

29. marital instability.tw,kw.

30. economic insecurity.tw,kw.

31. social inequit*.tw,kw.

32. legal problem*.tw,kw.

33. social welfare.tw,kw.

34. (environment* or air pollution).ti.

35. (green space* or blue space* or flood* or global warming*).tw,kw.

36. (lifestyle behav* or alcohol* or smok* or tobacco or drugs or narcotics).tw,kw.

37. (neighbourhood or neighborhood or voluntary work or volunteer* or unpaid work or loneliness or social isolation or social contact).tw,kw.

38. community.ti,kw.

39. (sleep or waking or insomnia*).tw,kw.

40. (physical* activit* or exercise or activ* travel* or walk or walking or cycling or running or jogging or gardening).tw,kw.

41. Loneliness/

42. exp Socioeconomic Factors/

43. Social Determinants of Health/

44. Race Factors/

45. Social Isolation/

46. Social Participation/

47. Housing/

48. Income/

49. Residence Characteristics/

50. Vulnerable Populations/

51. Social Support/

52. Diet/ae [Adverse Effects]

53. Food/ae [Adverse Effects]

54. Social Stigma/

55. Social Discrimination/

56. exp Family Relations/

57. Parents/ or Single Parent/

58. Prenatal Care/

59. Breast Feeding/

60. exp Child Abuse/

61. Violence/ or exp Domestic Violence/ or exp Intimate Partner Violence/

62. Crime/

63. Population Density/

64. exp Homeless Persons/

65. exp Educational Status/

66. Divorce/

67. Social Welfare/

68. exp Air Pollution/

69. Global Warming/

70. exp Life Style/

71. exp Alcohol Drinking/

72. exp Smoking/

73. exp Narcotics/

74. Drug Users/

75. Sleep/ or Sleep Deprivation/

76. Exercise/

77. exp Running/

78. Swimming/

79. Walking/

80. Gardening/

81. 1 or 2 or 3 or 4 or 5 or 6 or 7 or 8 or 9 or 10 or 11 or 12 or 13 or 14 or 15 or 16 or 17 or 18 or 19 or 20 or 21 or 22 or 23 or 24 or 25 or 26 or 27 or 28 or 29 or 30 or 31 or 32 or 33 or 34 or 35 or 36 or 37 or 38 or 39 or 40 or 41 or 42 or 43 or 44 or 45 or 46 or 47 or 48 or 49 or 50 or 51 or 52 or 53 or 54 or 55 or 56 or 57 or 58 or 59 or 60 or 61 or 62 or 63 or 64 or 65 or 66 or 67 or 68 or 69 or 70 or 71 or 72 or 73 or 74 or 75 or 76 or 77 or 78 or 79 or 80

82. mental health.tw,kw.

83. psychological* distress*.tw,kw.

84. mental* distress*.tw,kw.

85. mental* ill*.tw,kw.

86. depressive disorder*.tw,kw.

87. stress*.ti.

88. (anxiety or anxious).ti.

89. (lack* adj control).tw,kw.

90. depression.ti,kw.

91. emotional* strain*.tw,kw.

92. self-harm*.tw,kw.

93. suicid*.tw,kw.

94. psychological health.tw,kw.

95. (wellbeing or well being).ti,kw.

96. personality dysfunction.tw,kw.

97. (psychotic disorder* or psychosis).tw,kw.

98. PTSD.ti,kw.

99. mental* disorder*.tw,kw.

100. mental disease*.tw,kw.

101. psychological problem*.tw,kw.

102. psychiatric disorder*.tw,kw.

103. psychosocial problem*.tw,kw.

104. emotional health.tw,kw.

105. psychiatric* distress*.tw,kw.

106. Mental health/

107. Mental fatigue/

108. Mentally Ill Persons/

109. Stress, Psychological/

110. Anxiety/

111. Happiness/

112. "personal satisfaction"/

113. Mental disorders/

114. Depression/

115. Stress Disorders, Post-Traumatic/

116. exp Self-Injurious Behavior/

117. 82 or 83 or 84 or 85 or 86 or 87 or 88 or 89 or 90 or 91 or 92 or 93 or 94 or 95 or 96 or 97 or 98 or 99 or 100 or 101 or 102 or 103 or 104 or 105 or 106 or 107 or 108 or 109 or 110 or 111 or 112 or 113 or 114 or 115 or 116

118. 81 and 117

119. limit 118 to "reviews (best balance of sensitivity and specificity)"

120. exp animals/ not humans.sh.

121. limit 119 to (english language and yr="2000 - 2019")

122. 121 not 120

123. Public Health/

124. Policy-Making/

125. Health Policy/

126. Program Evaluation/

127. Organizational Policy/ or Public Policy/

128. Policy/ or exp Government/

129. ((national* or countr* or region* or government*) adj4 (strateg* or policy* or policies or program* or intervention* or initiative* or framework* or action* or plan* or approach*)).tw,kw.

130. 123 or 124 or 125 or 126 or 127 or 128 or 129

131. 122 and 130
