# Supplementary material for: National or population level interventions addressing the social determinants of mental health – an umbrella review
Source: BMC Public Health. 2021 Nov 18;21:2118. doi: 10.1186/s12889-021-12145-1 (PMC8599417; doi:10.1186/s12889-021-12145-1)
Supplement: Supplementary file 3 — Additional file 3. Data extraction tool [file 12889_2021_12145_MOESM3_ESM.docx]

| **Unique ID (1^st^ author and year)** |  |
| --- | --- |
| **Reference** |  |
| **Type of determinant (housing, welfare, employment etc.)** |  |
| **Extracted by (initials)** |  |
| **Date extracted** |  |
| **Review characteristics** | |
| • Key population-level determinants identified in review and their conceptual synonyms |  |
| • Is this a strategy, policy or intervention? (briefly describe) |  |
| - How is exposure to the determinant measured? |  |
| • MH outcome measures used (specified by review rather than results found) | At the individual level |
|  | At the population level |
| • Population |  |
| • Setting (Low, Middle or High Income countries) |  |
| • Number of studies included in review |  |
| • Number of professional/peer reviewed databases searched and disciplines |  |
| • Was grey literature search, contact with authors or citation follow-up carried out? (which one?) Include grey literature sources. |  |
| • Types of studies included |  |
| • Synthesis methods—narrative/meta-analysis/realist/etc. |  |
| • Time/language restriction |  |
| **Results of review** | |
| • Main results and strength of findings (size of effect) including variations by gender, ethnicity or socio-economic status  (Extract only relevant findings to the scope of our umbrella review (ie mental health outcomes, social determinants etc). Focus on review level findings not individual studies) |  |
| • Quality of underlying evidence: risk of bias and confounding, consistency across multiple settings (according to the review) |  |
| • Proposed mechanistic pathways ie how does the review propose that the social determinant influences mental health outcomes |  |
| • Clear evidence gaps identified |  |
| • Key contextual factors (if the review identifies political/social/historical or other factors which have influenced the effect of the social determinant, or which provide the background in which the social determinant acts) |  |
| - List any references (reviews of national social determinants interventions on MH) within the study reference list that should be considered for possible inclusion in our umbrella review (and flag to lead author) |  |
| **Quality checklist - Modified AMSTAR 2 (use other proforma and summarise findings here)** | |
| **Complete separate AMSTAR 2 checklist.**  **Any N/A in AMSTAR is fine. Look at supplementary data for review**  **Count weaknesses**  Critical weaknesses: Items 2, 4, 7, 9, 11, 13, 15 – partial yes still counts as critical weakness.  Non-critical weaknesses are all others (1, 3, 5, 6, 8, 10, 12, 14, 16)  **Guideline for grading see box 2 below**  For any queries with AMSTAR 2 questions see original AMSTAR 2 paper  <http://dx.doi.org/10.1136/bmj.j4008> | Critical weaknesses? (list which items) |
|  | Non-critical weaknesses? (list which items) |
|  | AMSTAR grading: High/medium/low/critically low confidence? |

| Box 2: Rating overall confidence in the results of the review |
| --- |
| • High   - No or one non-critical weakness: the systematic review provides an accurate and comprehensive summary of the results of the available studies that address the question of interest - •   Moderate - •   More than one non-critical weakness*: the systematic review has more than one weakness but no critical flaws. It may provide an accurate summary of the results of  the available studies that were included in the review - •   Low - •   One critical flaw with or without non-critical weaknesses: the review has a critical flaw and may not provide an accurate and comprehensive summary of the available studies that address the question of interest - •   Critically low - •   More than one critical flaw with or without non-critical weaknesses: the review has  more than one critical flaw and should not be relied on to provide an accurate and  comprehensive summary of the available studies  *Multiple non-critical weaknesses may diminish confidence in the review and it may be appropriate to move the overall appraisal down from moderate to low confidence. |
