# Supplementary material for: National or population level interventions addressing the social determinants of mental health – an umbrella review
Source: BMC Public Health. 2021 Nov 18;21:2118. doi: 10.1186/s12889-021-12145-1 (PMC8599417; doi:10.1186/s12889-021-12145-1)
Supplement: Supplementary file 4 — Additional file 4. Excluded studies at full text screening stage (with reason) [file 12889_2021_12145_MOESM4_ESM.docx]

**Umbrella Review – Excluded studies at full text screening stage, with reason (n=15)**

| **Reference** | **Reason for not including** |
| --- | --- |
| Ager, A. (2013). "Annual research review: Resilience and child well-being-Public policy implications." Journal of Child Psychology and Psychiatry **54**(4): 488-500. | Not a systematic review |
| Anderson, P., et al. (2012). "Alcohol and older people from a public health perspective." Annali dell'Istituto Superiore di Sanità 48(3): 232-247. | No outcome measure of MH |
| Bayer, J., et al. (2009). "Systematic review of preventive interventions for children's mental health: what would work in Australian contexts?" Australian & New Zealand Journal of Psychiatry 43(8): 695-710. | Not national level |
| Cook, K. (2012). "Neoliberalism, welfare policy and health: a qualitative meta-synthesis of single parents' experience of the transition from welfare to work." Health: an Interdisciplinary Journal for the Social Study of Health, Illness & Medicine 16(5): 507-530. | Not a systematic review |
| Corrigan, P., et al. (2015). "Do the effects of antistigma programs persist over time? Findings from a meta-analysis." Psychiatric Services 66(5): 543-546. | No outcome measure of MH |
| Corrigan, P. W., et al. (2012). "Challenging the public stigma of mental illness: A meta-analysis of outcome studies." Psychiatric Services 63(10): 963-973. | No outcome measure of MH |
| Dumesnil, H. and P. Verger (2009). "Public awareness campaigns about depression and suicide: a review." Psychiatric Services 60(9): 1203-1213. | No outcome measure of MH |
| Enns, J., et al. (2016). "Mapping interventions that promote mental health in the general population: A scoping review of reviews." Preventive Medicine: An International Journal Devoted to Practice and Theory 87: 70-80. | Not national level |
| Fiorati, R. C., et al. (2018). "Intersectorality and social participation as coping policies for health inequities-worldwide." Gaceta Sanitaria 32(3): 304-314. | No outcome measure of MH |
| Fisher, J. R. and M. Cabral de Mello (2011). "Using the World Health Organization's 4S-Framework to Strengthen National Strategies, Policies and Services to Address Mental Health Problems in Adolescents in Resource-Constrained Settings." International Journal of Mental Health Systems 5: 23. | Not a systematic review |
| Hillier-Brown, F., et al. (2019). "The effects of social protection policies on health inequalities: Evidence from systematic reviews." Scandinavian Journal of Public Health: 1403494819848276. | Not a systematic review |
| Johnston, V. (2009). "Australian asylum policies: have they violated the right to health of asylum seekers?" Australian and New Zealand Journal of Public Health 33(1): 40-46. | Not a systematic review |
| McCartney, G., et al. (2019). "Impact of political economy on population health: a systematic review of reviews." American Journal of Public Health 109(6): e1-e12. | Not a systematic review |
| Roy, M. J., et al. (2014). "The potential of social enterprise to enhance health and well-being: a model and systematic review." Social Science & Medicine 123: 182-193. | Not national level |
| Yonemoto, N., et al. (2019). "Implementation of gatekeeper training programs for suicide prevention in Japan: a systematic review." International Journal of Mental Health Systems 13: 2. | No outcome measure of MH |
