# Supplementary material for: National or population level interventions addressing the social determinants of mental health – an umbrella review
Source: BMC Public Health. 2021 Nov 18;21:2118. doi: 10.1186/s12889-021-12145-1 (PMC8599417; doi:10.1186/s12889-021-12145-1)
Supplement: Supplementary file 5 — Additional file 5. Summary table of included studies. [file 12889_2021_12145_MOESM5_ESM.docx]

**Umbrella review summary table of included reviews**

| **Review** | **Determinant** | **Brief description** | **No. of studies with mental health outcomes/ total** | **Mental health instruments or measures^[[1]](#endnote-1)^** | **Key findings** | **Mechanistic pathway suggested by authors** | **Quality of evidence AMSTAR^[[2]](#footnote-1)^** | **Contextual factors** | **Gaps in evidence identified** |
| --- | --- | --- | --- | --- | --- | --- | --- | --- | --- |
| **Demographic** | | | | | | | | | |
| Aitken et al, 2015 | Social welfare (paid maternity leave) | Association between paid maternity leave and maternal health (including mental health) at the individual and population level | 7/7 | CES-D, Kessler psychological distress scale | Positive association between paid maternity leave and mental health outcomes when measured at the individual level. | Key transition for women adapting to multiple roles, new identities, financial strain. Paid leave reduces this stress and aids physical and mental recovery | Critically low | Mainly high-income countries (except Lebanon) | Longitudinal studies |
| Borrell et al, 2014 | Gender equality policies (eg parental leave, reproduction) | The effect of gender equality policies on women’s health using between country or within country comparison | 3/19 | SF-36, Wellbeing index, CES-D, recorded mood and anxiety disorders | Reproductive policies, (mainly in US states) is associated with better mental health  Outcomes. Longer maternity leave associated with less depressive symptoms | Not described | Critically low | Mainly comparing US states | More diverse populations. More mental health outcomes |
| Staehelin et al, 2007 | Social welfare – length of maternity leave | The association between length of maternity leave and mental and physical health of mothers and children. | 4/13 | CES-D, Stewart, Spielberger, Mental Health Inventory, SOS | Longer maternity leaves are associated with fewer depressive symptoms and improved mental health | Maternity leave is part of maternity protection and benefits are presumed to include women’s and child health | Critically low | Limited to more educated populations in Europe and the US | Longitudinal evidence |
| **Economic** | | | | | | | | | |
| Egan et al, 2007 | Privatisation of public sector industries | Effect of privatisation on employee mental and physical health | 3/11 | Occupational Stress Index; self-report of psychiatric morbidity | Privatisation is associated with worsening employee mental health | Anticipated job insecurity and redundancies as key potential mechanisms for mental health impacts | Critically low | UK and European studies only; 20-30 years old | Longitudinal and controlled study evidence |
| Gibson et al, 2017 | Welfare-to -work employment conditional benefits | Mental health outcomes of lone parents and their children who received welfare-to-work interventions | 12/12  Only 2-5 in meta-analysis | Adult:CES-D, CIDI, UM-CIDI. Child BPI,PBS, SDI, Child Behavior Checklist. | Initial decline in parental mental health. Years 4-6 after the intervention, parental MH and child MH had improved with small effect sizes | Welfare-to-work can stimulate greater employment and income (particularly within the first 4 years). | High | Largely USA based studies | Studies outside North America.  Larger and meaningful increases in income. |
| Lucas et al, 2008 | Social welfare for socioeconomically disadvantaged families. | Effect of welfare intervention (direct cash payments, positive taxation) on child mental and physical health and social outcomes | 5/9 | Positive Child Behaviour scale, Behaviour Problem Index, Loneliness and Social Dissatisfaction Questionnaire, Revised Children's Manifest Anxiety Scale. | No consistent effect observed on child mental health or emotional state. Review authors suggested that there was ‘no evidence of effect’ rather than ‘evidence of no effect | Parental psychosocial stress linking low income and health outcomes. Any conditionality on financial support/welfare should not increase parental stress. | High | High income countries only included. | Unconditional payments of larger monetary value. Long-term child health outcomes.  Larger studies. |
| McAllister et al, 2018 | Social Welfare - family policy, employment policy, income support and social insurance policy, area-based initiatives and education policy | Effect of named policies on mental health at individual and population level | 21/21 | WHO-5, GHQ-12, SF36 and 12, CES-D8, MHI-5, ASQ-SE, HBSC  Suicide statistics and psychiatric diagnoses at population level. | Gender inclusive welfare state and neighbourhood renewal in deprived areas improve MH outcomes in women. Austerity associated with increased MH inequalities and suicide rates. Generous welfare benefits and area-based initiatives reduced inequalities in MH. | Welfare state interventions alleviate financial pressures on women particularly, reducing gender inequalities in mental health outcomes | Critically Low | Evidence from Nordic countries, Western Europe, North America, Australia or New Zealand included in review. | Education policies. Little on area-based initiatives and inequalities.  Natural experiments |
| O’Campo et al, 2015 | Welfare - Unemployment insurance | Level of unemployment insurance on mental and physical health, equity and social outcomes at individual and population level. | 22/33 | Not defined in review: outcomes relating to mental health and mental wellbeing in primary studies included | Generous UI benefits associated with better MH for unemployed and employed. Generous UI associated with re-employment being delayed to the end of the benefit period. | Generous UI increases financial security which increases psychological wellbeing. Effects on employed are through reducing job insecurity as a chronic psychosocial stressor. | Critically Low | Middle and high income countries across Europe, US and Asia.  Availability of jobs, severance packages are influential | Studies examining mechanisms of effect. |
| Pega et al, 2013 | Welfare – in-work tax credits for families | Effect of in-work tax credits on mental and physical health and social outcomes for working age adults. | 1/5 | Number of self-reported bad mental health days in past 30 days | No effect of in-work tax credits on mental wellbeing/psychological distress. No evidence to assess effects on mental illness. | Increase in relative income position reduces psychological stress.  Income effects on health are mediated by material conditions and in turn social exclusion (physical and psychological mechanisms) | High | Evidence from US only. | Non-US settings. Study designs with strong control of confounders. |
| Renahy et al, 2018 | Welfare – unemployment Insurance | Effect of UI on mental and physical health and social outcomes for working age adults. | 7/12 | Not specified in review; appear to be wellbeing and mental health problems. | Protective effect of UI generosity on subjective well-being for both the unemployed and employed. | UI attenuates negative impacts of poverty and unemployment on mental health. Potential negative mechanisms via disincentive to work, stigma | Critically Low | High income countries.  Social perception and wider job market context likely to impact effectiveness | Other contexts.  Impact of social perceptions |
| **Neighbourhood** | | | | | | | | | |
| Anderson et al, 2003 | Housing - Mixed-income housing in low SES neighbourhoods ; rental assistance programmes in relatively affluent neighbourhoods | Effect of these interventions on physical and mental health and wellbeing of participating households. | 12/23 | Self-reported symptoms of depression and anxiety by household head | No studies comparing outcomes of mixed-income housing programmes.  Two studies suggest a limited reduction of symptoms of depression and anxiety of rental assistance | Positive impacts on MH through improvement in neighbourhood physical and social conditions; negative impacts through disruptions of social ties and social deterioration | Critically Low | US context only: low socioeconomic status but ethnically diverse in urban areas, families with children | Mixed-income housing interventions Different settings; barriers to implementation |
| Thomson et al, 2013 | Housing: Warmth improvements, rehousing / retrofitting, basic improvements, rehousing from slums | Changes in health, illness, or well-being related outcomes among the residents following the delivery of a discrete housing improvement programme | 9/39 | Self-reported wellbeing: multiple measures including SF-36, HADS, GHQ, | Beneficial impacts of warmth interventions on mental health.  Suggestive of rehousing and refitting, rehousing from slums, housing led neighbourhood renewal benefitting MH. | Inhibiting a key intermediary between poverty and poor health. Qualitative data revealed links via increased thermal comfort, increased space, reduced noise and increased housing satisfaction. | Moderate | Evidence from LMIC | Robust evaluation. Basic housing improvements in LMIC |
| **Environment** | | | | | | | | | |
| Juarez et al, 2019 | Migration - to high income countries | Effect of restrictiveness of policies on mental and physical health of migrants. | 13/19 | CES-D; HSCL-25; PTSD scores | More restrictive policies for entry and integration increase the risk of poor mental health. Protective documentation policy to safeguard undocumented migrants protected against poor mental health | Exclusionist contexts were worse for mental health (where migrants are absorbed into the community). Better levels of mental health were associated with integrationist contexts (where migrants afforded rights within the new community) | Low | Migrant to high income countries only | LMICs.  Other dimensions of  migration policy, e.g. education, housing, deportation procedures. |
| **Social/Cultural** | | | | | | | | | |
| Gunnell et al, 2017 | Access to highly hazardous pesticides | Effectiveness of pesticide regulation in reducing the incidence of pesticide suicides and  overall suicides. | 27/27 | Incidence or estimated incidence of suicide by pesticide poisoning | National bans of highly hazardous pesticides, (particularly organophosphate insecticides and paraquat) could reduce the incidence of pesticide-specific and overall suicides | Restricting access to highly lethal and commonly used suicide means leads to a reduction in suicide rates | Critically low | A high proportion of suicides must be attributable to pesticide poisoning | High burden countries for suicide, formal time trend analyses, sales restrictions and other approaches |
| Kato et al, 2015 | Family interventions including parenting programmes | Evaluate population interventions that  aimed to lower the prevalence of mental health problems among children | 10/22 | SDQ, CBCL, CES-D for Children, ECBI, WEMWBS | Several promising community-level family interventions that may be effective in addressing children’s and families’ mental health problems | Behavioural problems are likely to lead to secondary mental health problems, such as depression | Critically low | Focus not only on high-risk populations, but also to implement as early as possible | Interventions that modify mild behavioural problems to prevent future MH problems |
| Mann et al, 2005 | Suicide prevention: education, screening, treating psychiatric disorders, access to means, media reporting | The effectiveness of specific suicide-preventive  interventions and recommendations for future prevention programs and research | 93/93 | Completed and attempted suicide, suicidal ideation; help seeking  behaviour, prescribing rates | Physician and gatekeeper education and restricting access to lethal means were the most successful components of suicide prevention strategies | Education and awareness among patients and/or physicians lead to increasing appropriate anti-depressant prescribing, lower rates of untreated major depression and lower suicide | Critically low | None stated | Identifying components of a multifaceted programme, longer-term trends |
| Moeller- Saxone et al, 2015 | No single determinant specified | Effectiveness of mental health promotion interventions in Asia; suicide prevention interventions in China. | 0/15 met criteria | N/A | No national level interventions found. | N/A | Critically Low | Asian countries only specified in review. | Evaluating programmes delivered at scale with larger populations. |
| Torok et al, 2017 | Public awareness/education | How mass media campaigns can be optimized to prevent suicide, looking at  their global efficacy, and mechanisms for successful outcomes | 13/13 | “suicide literacy”  Suicide deaths, attempts and ideation | Lack of evidence for mass media campaigns is lacking, particularly effecting behavioural change. Evidence linking mass media campaigns with modest increases in suicide knowledge and help-seeking | To change behaviour by affecting decision-making processes at the individual level through message promotion, potentially before crisis occurs. | Critically low | Level of exposure, repeat exposure, and community engagement appear to be fundamental to success. | Other media such as social media.  More robust evaluations of mass media campaigns |
| Zalsman et al, 2016 | Access to lethal means, media reporting, healthcare factors | Updated evidence for the eﬀectiveness of suicide prevention interventions since 2005 | 164/164 | Completed or attempted suicide, or suicidal ideation.  Intermediate outcomes - help-seeking behaviour | Restricting access to lethal means and school based educational programmes are associated with a decrease in suicide.  Insuﬃcient evidence for screening in primary care, in general public awareness campaigns, gatekeeper training and media guidelines. | Not specified | Critically low | Not specified | Prevention of hanging. Chain of care and follow-up.  Uptake of training in different contexts |
| Zechmeister et al, 2008 | No single determinant specified | Economic evaluations in  mental health promotion and mental illness prevention. | 14/14 | Non-specific [Mental well-being or mental disorder as an outcome | None of the studies reviewed provided evidence about scaled-up policies or interventions to prevent mental health. | Not specified | Critically low | Context varies considerably due to the heterogenous nature of the interventions | cost effectiveness of suicide prevention programme.’ |

1. Abbreviations: CES-D= Center for Epidemiologic Studies Depression Scale ; CIDI= Composite International Diagnostic Interview; UM-CIDI= University of Michigan Composite International Diagnostic Interview; SF-36/12 = Short Form Health Survey 36/12 Item; BPI= Behavior Problems Index; PBS= Problem Behavior Scale of the Social Skills Rating System; SDI= The Survey Diagnostic Instrument of the Ontario Child Health Survey; WHO-5= World Health Organisation-5 item wellbeing scale, GHQ-12 General Health Questionnaire – 12 item , MHI-5=Mental Health Inventory-5 item, ASQ-SE = Ages and Stages Questionnaire:Social and Emotional; HBSC=Health Behaviour in School Aged Children; SDQ= Strengths and Difficulties Questionnaire, CBCL= Child Behaviour Checklist; ECBI=Eyberg Child Behavior Inventory, WEMWBS=Warwick Edinburgh Mental Wellbing Scale, HADS= Hospital Anxiety and Depression Scale; HSCL-25=Hopkins Symptom Checklist – 25 item [↑](#endnote-ref-1)
2. AMSTAR rating and statement on underlying quality of studies as assessed by review [↑](#footnote-ref-1)
